# Three Cases of Scurvy

**Published:** 1888-08

**Authors:** Frederick P. Henry

**Affiliations:** Physician to the Philadelphia Hospital, and to the Episcopal Hospital


### Three Cases of Scurvy.

BY FREDERICK P. HENRY, M.D., PHYSICIAN TO THE PHILADELPHIA HOSPITAL, AND TO THE EPISCOPAL HOSPITAL.

[Delivered at the Philadelphia Hospital, May 23, 1888.]

*Gentlemen:*—I have to show you this morning three cases of a disease of malnutrition, which, before the days of canned vegetables and fruits, was very prevalent, and is perhaps more common at present than is generally supposed. The disease to which I refer is scurvy, and it is one which has become almost obsolete—certainly obsolescent—by the removal of its causes. The principal, if not the sole cause of this disease, is a want of variety in the food. It was for a long time supposed that common salt taken in large quantities for a prolonged period, had a direct tendency to cause scurvy ; but this has been disproved by the observation of cases in individuals who had not eaten of salted food of any kind. The disease is now regarded as a species of starvation, or partial inanition, caused by the want of certain unknown substances found in potatoes, green vegetables, lemons, oranges, and limes. Perhaps the most recent epidemic of scurvy of which we have any scientific account, occurred during the siege of Paris by the Germans in 1870–71, and it was observed in several of the large hospitals of that city, that the disease made its appearance immediately after the enforced withdrawal from the diet of potatoes and green vegetables. Numerous instances of the same sort may be found in the literature of this disease.

Dr. Garrod in 1848, was the first to propound the theory that the substance, the absence of which from the food gives rise to scurvy, is potassium ; and this view of the etiology of the disease has been widely accepted, numbering among its adherents the great chemist, Liebig. The great antiscorbutic value of potatoes is due to the potassium, which it contains in large amount. That the absence of potassium from the food is one of the causes of scurvy, there can be no doubt, but the view that it is the sole cause cannot be maintained; for epidemics have been observed among individuals into whose dietary potassium entered in large amount. The obscurity which surrounds the etiology of this disease, taken in connection with the fact that it frequently prevails in epidemic form, has, as a matter of course, given rise to the suspicion that it originates from, and is propagated by, infective agencies—micro-organisms. This hypothesis has not the slightest basis of fact upon which to stand, and is, as I have said, nothing more than a bare suspicion. There is, however, one fact which has seemed to lend some support to this view, viz : that the disease has been observed in infants at the breast, and the breast has been always that of a scorbutic mother. It is hardly necessary to say that a poorer milk can be scarcely conceived of than that secreted by a scorbutic woman, and this fact of the occurence of scurvy in nursing children is, in reality, a strong confirmation of the view that the disease is one of defective alimentation.

Predisposition.—That there is a predisposition to this disease is shown by the fact, that in epidemics of scurvy a certain number of persons escape who have been living on precisely the same food as those attacked. The strong, as is to be expected, resist the disease better than the weak, while those who have recently suffered from intermittent fever or syphilis, are especially vulnerable. Bad hygienic surroundings, excessive physical labor and depressing emotions are undoubted predisposing causes. It is probable that the first two named causes were operative in our patients, who all belong to the laboring class. Two of them are Hungarians who have been working in a stone quarry, and the third is an Italian. None of them speak a word of English, and, therefore, it has been difficult to obtain precise details concerning their diet and mode of life. The Italian, doubtless, has been living largely on starchy food, macaroni or rice, both of which are very deficient in potash. Of sixteen alimentary substances arranged by Garrod, with reference to the amount of this substance contained in them, potatoes head the list and rice comes last.

Clinical History.—That scurvy is a constitutional, and not a local disease is shown by the fact that its pathognomonic externternal manifestations are preceded by a variable period of ill health or cachexia, of which the symptoms are largely identical with those of anæmia in general. In some respects, however, they are decidedly unlike those of anæmia. For instance, the countenance, instead of being pale, is of a livid, cyanotic hue, as if the blood were not thoroughly decarbonized, and it is said that direct observation has shown the blood, at this stage, to contain more than the normal percentage of coloring matter. Be this as it may, and I have had no opportunity to confirm or refute the statement, it is certain that anæmia is sure to occur sooner or later. In the patient before you, a Hungarian, who has been in the hospital for several weeks, I examined the blood on May 16, and found it to contain 2,775,000 red corpuscles per cubic millimetre, *i. e.*, 55 per cent. of the normal number, while the coloring matter was only 30 per cent. of the normal. These figures represent a very high degree of anæmia. In the second of the patients, the Italian, I examined the blood on May 22, and found a still higher grade of anæmia. The red corpuscles in this case numbered only 1,980,000 *i. e.*, their per cent. was only 39. After repeated attempts to obtain blood for the color test, I was obliged to desist, although several deep incisions with a broad lance-shaped needle were made in three separate fingers. The blood would at first flow readily and then stop altogether, before a good-sized drop had escaped. It seemed as if all the blood in the finger-pulp had been squeezed out for no more could be obtained. The white corpuscles were certainly not increased in either case, and in the latter I believe they were not only relatively, but absolutely diminished, as I examined a great many fields without finding any, and at last, after a prolonged search, succeeded in finding *one*.

After the prodromic stage of cachexia has lasted for a period, ranging from one to several weeks, the true nature of the disease becomes manifest by the appearance of certain local affections, ot which one of the earliest, and certainly the most characteristic is that of the gums. This is well marked in all three of our cases. The gums, as you see, are of a deep-red color and are swollen to such an extent that they project in fungous masses between the teeth. They are very painful and tender, and bleed on slight pressure. They may swell to such an extent as to entirely cover the teeth, or actually to protrude from the lips.

It is a singular fact that the affection of the gums is limited to the immediate neighborhood of the teeth. If there is a gap between them the corresponding portion of the gums remains healthy, and, therefore, in infants who have not yet cut their teeth, and in old people whose teeth, so to speak, have *cut* them, the affection of the gums is wanting. The other manifestations of scurvy have their principal seats in the skin, the connective tissues and the muscles. In the skin they may assume the form of small petechial spots surrounding a hair follicle, as is evidenced by the fact that their centre is perforated by a hair. This petechial eruption is present in typical form in the most recent of our cases. Sometimes the eruption appears in the form of blood-containing vesicles, which may be of such large size as to resemble the form of skin disease called pemphigus. The vesicle may rupture and its base ulcerate. Sometimes there may be large subcutaneous extravasations of blood. These eruptions, if such they may be called, are most common in the lower extremities, a fact exemplified in all three cases. In cases 1 and 2 there are considerable swelling and tenderness of both legs, which are covered with large bruise-like patches of extravasation. The largest patch is on the anterior surface of the right tibia and is several inches in length. It is of a deep purple color, and over it the epidermis is thick and desquamating. Extravasations into the connective tissue and muscles may give rise to great pain, swelling and loss of movement in the affected limb, generally one of the lower extremities. The calf of the leg, the popliteal space and the loose connective tissue around the tendo Achillis, are favorite sites of these extravasations, but the muscles of the thigh and trunk, such as the recti abdominis and the pectorales, may be attacked. The swellings may be at first soft, later, of a board-like hardness. The skin covering them sometimes inflames and suppurates, giving exit to grumous blood, and leaving behind an ulcerating cavity. Hemorrhage in other situations is quite common, especially in the form of epistaxis, which may be of daily or nightly occurrence. In case 1 epistaxis occurred every night. Hemorrhages from the bowels may be spontaneous, but are more apt to follow the admistration of purgatives. Hæmaturia sometimes occurs.

The effusion of pleurisy or pericarditis in a scorbutic individual will certainly be hemorrhagic. More than a month ago I exhibited in this room a patient from whose left pleural cavity I removed twenty-two ounces of hemorrhagic effusion. I said at the time that, in my opinion, a sanguinolent pleural effusion was not so much an indication of tuberculosis of the pleuria as of a scorbutic diathesis. This patient has since died, and at the autopsy, no tubercle was found in either lungs or pleura.

In severe cases the bones may be affected in various ways. Either spontaneously or as the result of slight injuries which, in a normal constitution, would be followed by no such effect, extravasations of blood take place beneath the periosteum which, if extensive, may lead to partial necrosis of the underlying bone. The tibia is the bone most frequently affected in this way, doubtless on account of its exposed situation. Sometimes the epiphysis of a bone is separated from its attachment to the shaft, by the extravasation of blood between the two portions. This is most frequently observed in the ribs at their point of junction with the costal cartilages. The first symptom of this complication is a painful and tender swelling at the point of junction of rib and cartilage, followed by abnormal mobility and crepitation of the affected spot. Several ribs on either side may be affected in this manner, the result of which may be that the sternum, deprived of its usual supports, sinks inward toward the spine. It is said that reunion of the separated portions will occur in case of recovery; but when the disease is accompanied with such changes as these, it generally ends fatally.

The callus uniting recent, or even very old fractures, is apt to soften in well-marked cases of scurvy. Inflammatory hemorrhagic effusions into the joints, especially of the knee and ankle, have been observed and are known as scorbutic arthritis.

A peculiar affection of vision, a form of amblyopia known as hemeralopia, has been observed in a number of cases. By hemeralopia is meant an inability to see distinctly except in a bright light. Another name for the affection is night-blindness. It has been most frequently observed in sailors who have been long exposed to the glare of a tropical sun while suffering from scurvy or greatly debilitated from an improper supply of food. It is said that the ophthalmoscopic examination of such cases does not yield any positive results. The disorder of vision is doubtless caused by exhaustion of the retina, which, in order to perform its function, requires the stimulus of a strong light.

Although probably no case of scurvy runs its course without abnormal rise of the body temperature, fever is not an essential feature of the disease, but is secondary to the inflammatory complications of the gums, skin, connective tissue, bones, joints, etc. In all three cases the temperature has been above the normal, and in one it was 103° at the time of admission.

The origin of this disease has naturally been sought in the blood, but thus far no definite change has been detected in the composition of this fluid; that is, no change not met with in other forms of anæmia not attended with scurvy. The conclusions of chemists with reference to the composition of the blood are diametrically opposite. Thus one set of chemists has demonstrated an increase of the salts of the serum, especially the chloride of sodium, to double the normal amount, while another has been able to prove that they are diminished. A more definite result, certainly none could be more indefinite, might be reached by the analysis of the solid tissues, especially the blood vessels, connective tissue and muscles.

TREATMENT.—If diseases were divided into preventable and non-preventable, scurvy would certainly head the list of the former, for there is none concerning the prophylaxis of which we have more certain rules. Without entering into particulars, which may be readily inferred from what I have said of the causes of this disease, it may be asserted that scurvy will never make its appearance, either in laboring or idle men, who are supplied with plenty of nutritious food. From an anti-scorbutic standpoint no single article of food can be compared with the potato. In the treatment of the disease itself, immediate attention should be paid to the gums; for until they are in a comparatively healthy state, the first two steps in the digestion of solid food, mastication and insalivation, cannot be taken. While life may be supported indefinitely by milk, eggs, and soup, such life is not one of hard work. Solid food is the prerequisite of solid work.

Gargles of solutions of potassium chloride and tincture of myrrh will be found of service; also, painting the gums with strong solution of nitrate of silver, or with the solid stick. The strong fetor of the breath may be corrected by adding carbolic acid to the mouth wash. Two or three grains to the ounce will be sufficient. Internally, lemon juice should be administered. One of our patients has greatly improved while consuming six lemons daily. If iron is tolerated, it may be administered in the form of Blaud's pill, which contains potassium as well as iron.

